# Immortalization up‐regulated protein promotes tumorigenesis and inhibits apoptosis of papillary thyroid cancer

**DOI:** 10.1111/jcmm.16018

**Published:** 2020-10-23

**Authors:** Lizhi Lin, Jialiang Wen, Bangyi Lin, Adheesh Bhandari, Danni Zheng, Lingguo Kong, Yinghao Wang, Ouchen Wang, Yizuo Chen

**Affiliations:** ^1^ Department of Thyroid and Breast Surgery The First Affiliated Hospital of Wenzhou Medical University Wenzhou PR China

**Keywords:** apoptosis, biomarker, Hippo‐YAP1 pathway, immortalization up‐regulated protein, papillary thyroid cancer, progression

## Abstract

The incidence of thyroid cancer is increasing in recent years worldwide, but the underlying mechanisms await further exploration. We utilized the bioinformatic analysis to discover that Immortalization up‐regulated protein (IMUP) could be a potential oncogene in the papillary thyroid cancer (PTC). We verified this finding in several databases and locally validated cohorts. Clinicopathological features analyses showed that high expression of *IMUP* is positively related to malignant clinicopathological features in PTC. Braf‐like PTC patients with higher *IMUP* expression had shorter disease‐free survival. The biological function of *IMUP* in PTC cell lines (KTC‐1 and TPC‐1) was investigated using small interfering RNA. Our results showed that silencing *IMUP* suppresses proliferation, migration and invasion while inducing apoptosis in PTC cell lines. Changes of the expression of apoptosis‐related molecules were identified by real‐time quantitative polymerase chain reaction and Western blotting. We also found that YAP1 and TAZ, the critical effectors in the Hippo pathway, were down‐regulated when the *IMUP* is silenced. Rescue experiments showed that overexpression of *YAP1* reverses the tumour inhibitory effect caused by *IMUP* knockdown. Our study demonstrated that *IMUP* has an oncogenic function in PTC and might be a new target gene in the treatment of PTC.

## 1 INTRODUCTION

Thyroid cancer (TC) is the most prevalent malignant disease of the endocrine system, of which incidence has been increasing over recent years. In 2020, there are more than fifty‐thousands of estimated new cases occur in the USA, ranking the fifth highest of all carcinoma types in women, along with about two‐thousands estimated death cases.^1^ The same trend could also be seen in China, though the mortality rate remains low, TC has become one of the fastest increasing malignant diseases.^2^ Papillary thyroid cancer (PTC) takes up almost 85% of all thyroid cancer cases.^3^ Meanwhile, the rising incidence rate and mortality of PTC contribute to the actual increase of all TC cases.^3^, ^4^ Almost 50% of PTC patients appear with positive lymph nodes, and 68% of high‐risk differentiated thyroid cancer patients could have persistent structural disease or recurrence after a standard regimen.^5^, ^6^ These observations motivated us to explore the underlying mechanisms of PTC further.

Previous studies have shown that the majority of mutations in PTC are associated with mitogen‐activated protein kinase (MAPK) signalling pathway, such as *BRAF* and *RAS* genes. Novel low‐frequency mutations such as *EIF1AX*, *PPM1D* and *CHEK2* are discovered to reclassify TC into different molecular subtypes.^7^ In our study, we found that Immortalization up‐regulated protein (IMUP) could be associated with the progression and development of PTC.

*IMUP* is also known as Hepatocyte Growth Factor Activator Inhibitor Type 2‐Related Small Protein (*H2RSP*) or *C19orf33*. This protein‐coding gene locates on chromosome 19q13.2. Previous studies showed that *IMUP* is involved in SV40‐mediated immortalization in human fibroblasts and is also related to tumorigenicity and cellular proliferation.^8^, ^9^ In the area of cancer research, *IMUP* was up‐regulated in endometrial carcinoma and its higher expression was associated with the aggressive features of breast tumours.^10^, ^11^ However, no investigation has been done regarding the biological function of *IMUP* in PTC yet.

In the present study, we validated that *IMUP* is significantly higher in PTC tumour tissues compared with matched normal tissues, examined the relationship between PTC clinical characteristics and *IMUP* expression. We performed a serial of experiments to reveal the effect of IMUP silencing on PTC cell lines, as well as the relationship between the *IMUP* expression and the Hippo pathway markers such as *YAP1*. Our results implied that the *IMUP* could be a crucial oncogene and might be a potential target for the therapy of PTC in the future.

## 2 MATERIALS AND METHODS

### 2.1 Bioinformatics analysis

The mRNA expression data were downloaded from The Cancer Genome Atlas (TCGA) data portal (https://tcgadata.nci.nih.gov/tcga/) and Gene Expression Omnibus (GEO) database (https://www.ncbi.nlm.nih.gov/gds/). Transcriptome sequencing data of 502 PTC tissues with matched complete clinicopathological characteristics data and 58 non‐tumour tissues were collected from TCGA. The GSE33630, GSE60542 and GSE35570 data sets were based on the GPL570 platform, Affymetrix Human Genome U133 Plus 2.0 Array, merged and processed by robust multi‐array average (RMA) then normalized using the quantile normalization. The GSE50901 database was based on the GPL13607 platform, Agilent‐028004 SurePrint G3 Human GE 8x60K Microarray, numeric data were generated and normalized by intensity‐dependent global normalization (LOWESS). In total, 170 PTC tumour samples with associated clinicopathological features and 127 non‐tumour samples were selected. The volcano plot was generated using the Limma R and ggplot package to present the differential expression genes (DEGs) between cancerous tissues and normal tissues in corresponding databases. Kaplan‐Meier plots for disease‐free survival (DFS) were obtained from the Gene Expression Profiling Interactive Analysis 2 (GEPIA2) (http://gepia2.cancer‐pku.cn). Gene Set Enrichment Analysis (GSEA) was conducted using GSEA v3.0 software (http://www.broadinstitute.org/gsea), which analysed the differences in mRNA expression levels of biological annotation and pathways to discover the downstream signalling pathway of *IMUP*.

### 2.2 Patients and specimen collection

Thirty‐nine pairs of PTC tissues and matched adjacent normal tissues were collected from patients who had initial surgery at the Department of Thyroid and Breast Surgery, The First Affiliated Hospital of Wenzhou Medical University. All samples were snap‐frozen in liquid nitrogen and stored at −80°C before RNA extraction. Informed consent was obtained from each patient for the scientific use of specimens. Procedures performed in our study were approved by and conducted following the ethical standards of the Institutional Review Board of First Affiliated Hospital of Wenzhou Medical University (approval no. 2012‐57). Two senior pathologists confirmed all histological diagnoses by retrospectively reviewing tumour specimens. The *IMUP* expression from RNA‐seq data of 70 pairs of PTC and matched normal tissues was obtained from our unpublished data.

### 2.3 Cell lines and cell culture

KTC‐1, TPC‐1 and BCPAP cell lines were provided by Prof. Mingzhao Xing of the Johns Hopkins University School of Medicine (Baltimore, MA, USA). HITORI3 was obtained from the Cell Bank of the Shanghai Chinese Academy of Sciences (Shanghai, China). The PTC cell lines were cultured in RPMI‐1640 containing 10% foetal bovine serum (FBS) (Gibco, Invitrogen, Carlsbad, CA, USA). All cell lines were incubated under an atmosphere of air containing 5% CO_2_ at 37°C.

### 2.4 RNA extraction and real‐time quantitative polymerase chain reaction (qRT‐PCR)

RNA from the patients’ tumour specimens and cell lines was extracted using TRIzol reagent (Thermo Fisher Scientific, Waltham, USA) in compliance with the manufacturer's protocol. The quality (A260/A280 ratios) and quantity of the extracted RNA were assessed using spectrophotometry NanoDrop 1000 (Thermo Fisher Scientific). Reverse transcription reaction was performed using ReverTra Ace qPCR RT Kit (Toyobo, Osaka, Japan) following the manufacturer's protocol (20 μl reaction; 1000 ng of total RNA; step 1 16°C for 5 minutes, step 2 42°C for 30 minutes, step 3 98°C for 5 minutes). cDNA was stored at −20°C. The PCR analysis was performed using the Applied Biosystems 7500 Real‐Time PCR system (Applied Biosystems, Thermo Fisher Scientific, Inc) and the SYBR Premix Ex Taq II kit (RR820A, TaKaRa, Dalian, China) in compliance with the manufacturer's protocol in triplicate. The relative expression of mRNA was calculated using the comparative cycle threshold (2^−ΔΔCT^) method with GAPDH used as the endogenous control. The primer sequences were used as follows: *IMUP* forward‐GGTTTAATGAGCCCTGTCC/reverse‐CAAGAAGCCCAAAGTGAAGA; *BCL‐2* forward‐GACTTCGCCGAGATGTCCAG/reverse‐GAACTCAAAGAAGGCCACAATC; *BAX* forward‐CGAACTGGACAGTAACATGGAG/reverse‐CAGTTTGCTGGCAAAGTAGAAA; *CASPASE‐8* forward‐ACATGGACTGCTTCATCTGC/reverse‐AAGGGCACTTCAAACCAGTG; *CASPASE‐9* forward‐TGCTGCGTGGTGGTCATTCTC/reverse‐CCGACACAGGGCATCCATCTG; *YAP1* forward‐AGAACAATGACGACCAATAGCTC/reverse‐GCTGCTCATGCTTAGTCCAC.

### 2.5 Cell transfection

Thyroid cancer cells were seeded into 6‐well plates and cultured for 1 day before transfection (About 6 × 10^4^ TPC‐1 cells per well and 8 × 10^4^ KTC‐1 cells per well). The *IMUP* expression was silenced by small interfering RNA (siRNA) mixed with Lipofectamine RNAiMAX transfection reagent (Invitrogen, Grand Island, NY, USA) according to the manufacturer's protocol. Plasmids of pcDNA3.1 and pcDNA3.1‐YAP1 were purchased from GenePharma (Shanghai, China), and transfection was conducted using the Lipofectamine 3000 reagent (Invitrogen). The medium was replaced 7 hours after transfection. Cells were harvested 48 hours after transfection for the following analyses and assays. All siRNAs were obtained from GenePharma. The siRNA sequences used in the study were as follows: SiRNA1, sense (5′‐3′) GGUCCGGGUCCAAAGCAAGTT, antisense (5′‐3′) CUUGCUUUGGACCCGGACCTT; SiRNA2, sense (5′‐3′) GGAUGUGAAGUCCCACGCUTT and antisense (5′‐3′) AGCGUGGGACUUCACAUCCTT.

### 2.6 Cell proliferation and colony formation assay

Cell counting kit‐8 (CCK‐8) assay was used to evaluate cell proliferative capacity. TPC‐1 and KTC‐1 cells that had been transfected were plated into 96‐well plates (1250 TPC‐1 cells per well and 1500 KTC‐1 cells per well). Then, the cells were incubated with the 10 ml CCK‐8 solution (Beyotime Biotechnology, Shanghai, China) at 37°C for 2‐4 hours. The absorbance at 450 nm was quantitated by spectrophotometer on four consecutive days. As for the colony formation assay, the same number of cells was seeded into 6‐well plates and incubated for 7 days in the atmosphere as above. Then, the cells were fixed with 4% paraformaldehyde for 30 minutes and stained with 0.1% crystal violet solution for 30 minutes at 37°C. Images were captured by a digital camera.

### 2.7 Cell migration and invasion

Transwell chambers (#3422, Corning, NY, USA) were used in the cell migration assays. TC cells (3.5 × 10^4^ cells/well) were transferred into the upper chamber in the serum‐free medium after transfection. The lower chamber contained 0.6 ml medium supplemented with 10% FBS. Cells were incubated for 22 hours before the low chamber cells being fixed with 4% paraformaldehyde and stained with 0.4% crystal violet solution. Cells that were not able to migrate to the lower chamber were carefully removed. Five stochastic fields of view were captured by the digital camera under the microscope at ×20 magnification for analysis. The invasion ability was examined using the Matrigel invasion chamber (#354480; Corning Biocat, NY, USA) with the same procedure as described. Scratch wound experiments were conducted in 24‐well plates; 2.0 × 10^5^ cells were incubated with serum‐free culture for 48 hours after wounded with a plastic tip. The gaps and cells were imaged before and after scratching, and images were captured by a digital camera under the microscope at ×5 magnification. The wound healing area was calculated using Image J software (NIH, MD, USA). The migrating rate (%) = (wound area at 0 h–wound area at 48 h)/wound area at 0 h × 100%.

### 2.8 Flow cytometry

TPC‐1 and KTC‐1 were seeded into 6‐well plates and incubated for 24 hours. Then, the cells were transfected by siRNA or plasmids before incubated for another 48 hours. Transfected cells were collected and washed three times with phosphate‐buffered saline, then were resuspended in 500 μl 1 × binding buffer at a concentration of 1.0 × 10^6^ cells/ml. The Annexin V‐FITC/propidium iodide (PI) apoptosis kit (Becton, Dickinson and Company, Franklin Lakes, NJ, USA) was used to determine the apoptotic proportion of transfected TPC‐1 and KTC‐1 cells according to the manufacturer's protocol. Cell suspensions of 500 μl were stained with 5 μl Annexin V‐fluorescein isothiocyanate and 5 μl PI at room temperature for 15 minutes in the dark. All data were analysed by flow cytometry (BD Biosciences AccuriC6, Becton, Dickinson and Company, Franklin Lakes, NJ, USA) and Flowjo software (FlowJo).

### 2.9 Western blot analysis

RIPA lysis buffer (Solarbio, Beijing, China) was used to lyse transfected cells, while phenylmethylsulphonyl chloride was used to prevent degradation of the protein. Bicinchoninic acid (BCA) assay (Thermo Scientific, USA) was used for quantification of the protein. Then, each sample was separated by sodium dodecyl sulphate‐polyacrylamide gel electrophoresis (BioRad, Berkeley, CA, USA) and transferred to polyvinylidene difluoride (PVDF) membranes (EMD Millipore, Billerica, MA, USA). PVDF membranes were blocked by 5% non‐fat milk for two hours at room temperature and then incubated with primary antibodies overnight at 4°C. Primary antibodies were as follows: IMUP, BCL‐2, BCL‐XL (Abcam, Cambridge, MA, USA); YAP1, TAZ, BAX, Cleaved‐Caspase9 and *β*‐Actin (Proteintech, Wuhan, China). After being washed by TBST (Tris‐buffered saline/ 0.1% Tween 20), the membrane was incubated with anti‐rabbit IgG or anti‐mouse IgG (1:5000. Abcam, Cambridge, MA, USA) for two hours at room temperature. Finally, the chemiluminescence kit (Thermo Scientific, USA) was used to visualize the blots, and images of the results were analysed with ImageJ software (NIH).

### 2.10 Statistical analysis

SPSS software version 22.0 (IBM SPSS Inc, Chicago, IL, USA) and Graphpad Prism8 software (GraphPad, CA, USA) were used for all statistical analyses. Data displayed normal distribution was analysed by Student's *t* test. Data with non‐normal distribution were analysed by the Mann‐Whitney test and the Wilcoxon test. Each categorical variable was analysed by χ^2^ tests. Logistic regression analysis was performed for the prediction of lymph node metastasis. The CCK‐8 assay was analysed by two‐way ANOVA. The Kruskal‐Wallis test was used for comparisons of data with more than two group. The receiver operating characteristic (ROC) curve was applied to evaluate the diagnostic efficacy of *IMUP* in PTC, and *P* < 0.05 was considered to indicate a statistically significant difference.

## 3 RESULTS

### 3.1 Identification of DEGs in microarray analysis

**FIGURE 1:**
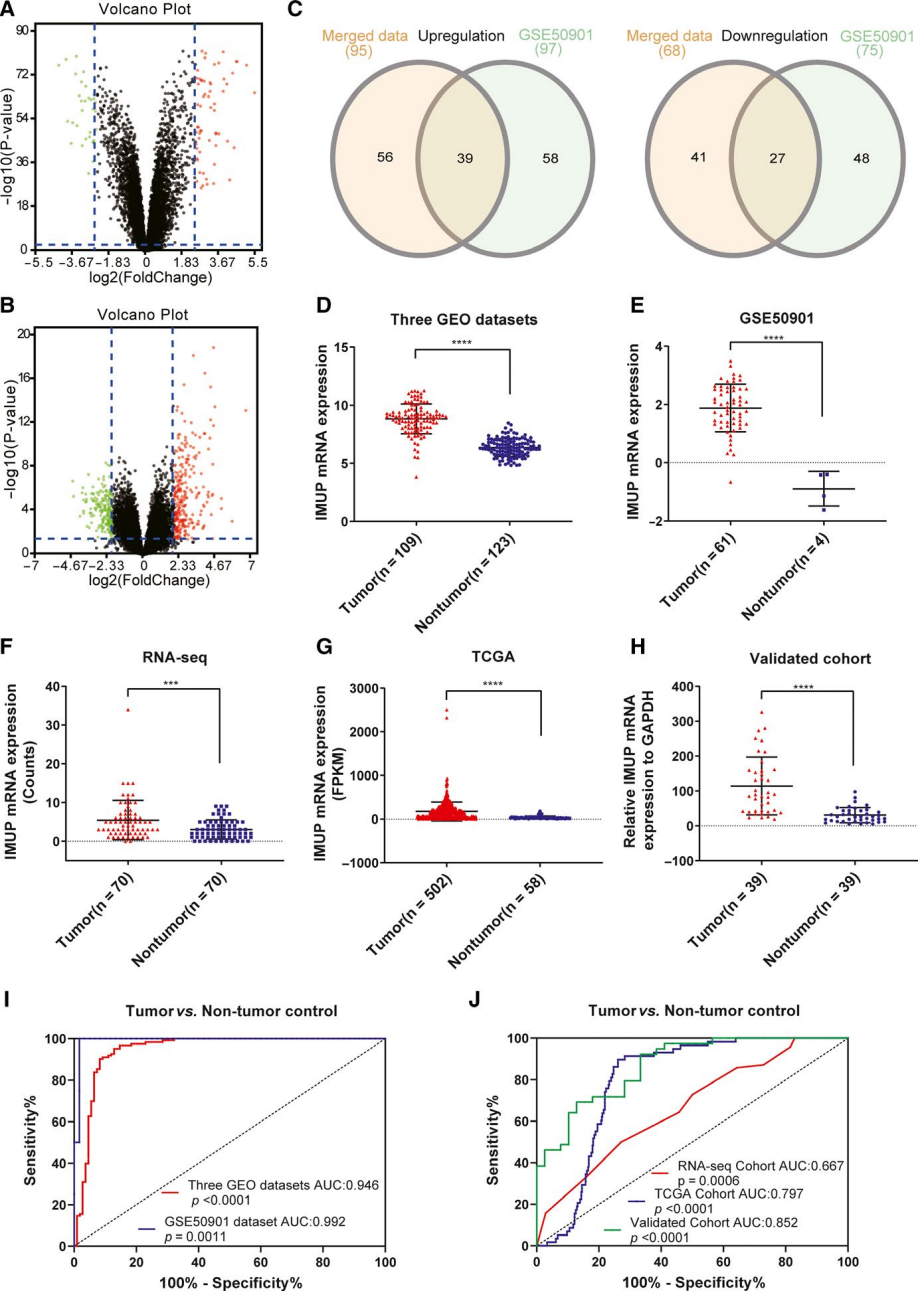
*IMUP* is up‐regulated in PTC. (A) The volcano plot in integrated three GEO datasets (log2[|FC|] ≥ 2.5, *P* < 0.01). (B) The volcano plot in GSE50901 datasets (log2|FC| ≥ 2, *P* < 0.05). (C) Venn diagram of the collection of up‐regulated genes and down‐regulated genes in the three integrated data sets and GSE50901. The expression of the *IMUP* gene in PTC tumour was significantly higher than non‐tumour tissues in the merged GEO data set (D), in the GSE50901 data set (E), in our RNA‐seq data (F), in the TCGA cohort (G), in the validated cohort using qRT‐PCR (H) (FPKM: Fragments per kilobase per million). Statistical analyses were performed as follows: [D, G]: Mann‐Whitney test; [E]: Student's *t* test; [F, H]: Wilcoxon test; ***P < 0.001, ****P < 0.0001. (I) ROC curve for expression of *IMUP* to diagnose PTC in the merged GEO data set and the GSE50901 dataset. (J) ROC curve for expression of *IMUP* to diagnose PTC in the RNA‐seq data, the TCGA database and our validated cohort

To study the differences in mRNA expression between PTC tumour tissues and non‐tumour tissues, we extracted data from the GEO databases, including GSE33630, GSE60542, GSE35570 and GSE50901 ^12^, ^13^, ^14^, ^15^ (Table S1). Data from GSE33630, GSE60542 and GSE35570 were using the same platform, so they were integrated and normalized for further analyses. The expression data before and after normalization with the RMA method were shown in Figure S1A,B. We then screened the integrated data using the Limma R package (log2|fold change (FC)| ≥ 2.5, *P* < 0.01), identifying 163 DEGs, consisting of 95 up‐regulated genes and 68 down‐regulated genes (Figure 1A). Sample data from the GSE50901 database were also screened (log2|FC| ≥ 2, *P* < 0.05), identifying 172 differentially expressed genes with 97 up‐regulated genes and 75 down‐regulated genes (Figure 1B). Then, we obtained 39 common up‐regulated genes and 27 down‐regulated genes from two sets using the Venn diagram (Figure 1C).^16^

### 3.2 *IMUP* is up‐regulated in the PTC tumour

Among all DEGs, the gene *IMUP* drew our attention. In microarray analysis, we found that the *IMUP* expression level is significantly higher in PTC tumour tissues than non‐tumour tissues (Figure 1D: Three integrated GEO dataset, *P* < 0.0001; Figure 1E: GSE50901, *P* < 0.0001). We verified the up‐regulation of *IMUP* in PTC from our RNA sequencing data (*P* < 0.001, Figure 1F) and TCGA database (*P* < 0.0001, Figure 1G). Besides, we also validated the result by performing qRT‐PCR on 39 paired PTC tissues and matched normal tissues (*P* < 0.0001, Figure 1H). To identify the diagnostic value of highly expressed *IMUP* in PTC tumour, we conducted ROC curve analyses, producing an area under the curve (AUC) of 94.6% for the three integrated three GEO datasets (95% confidence interval [CI]: 91.2%‐98.0%, *P* < 0.0001), 99.2% for the GSE50901 dataset (95% CI: 97.2%‐100.0%, *P* = 0.0011), 79.7% for TCGA cohort (95% CI: 75.7%‐83.7%, *P* < 0.0001), 85.2% for validated cohort (95% CI: 79.4%‐94.5%, *P* < 0.0001), 66.7% for the RNA‐seq cohort (95% CI: 57.9%‐75.6%, *P* = 0.0006) (Figure 1I‐J). All these findings indicated that the gene *IMUP* is up‐regulated in PTC and might be a diagnostic biomarker.

### 3.3 The association between *IMUP* expression and clinical features of PTC patients

**FIGURE 2:**
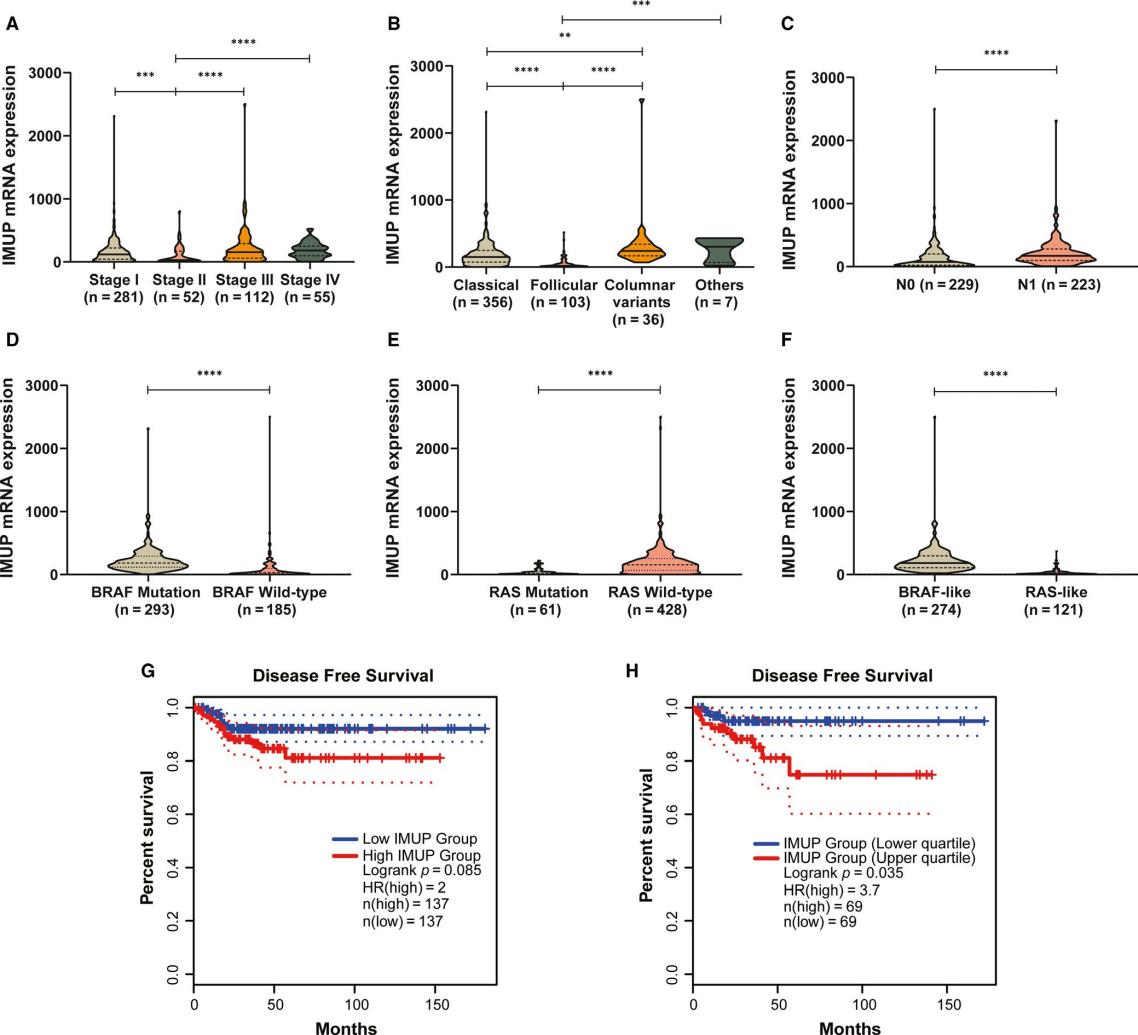
*IMUP* expression is correlated with various clinical features and prognosis of patients with PTC. (A) The expression of *IMUP* differed in different tumour stages. Higher *IMUP* expression tended to be associated with a more advanced tumour stage. (B) The expression of *IMUP* in different subtypes of PTC *IMUP* was significantly up‐regulated in the classical subtype and the columnar variant subtype compared with the follicular subtype. (C) *IMUP* expression was significantly higher in the lymph node metastasis‐positive group than the lymph node metastasis‐negative group. (D) *IMUP* expression in the BRAF mutation group was higher than in the BRAF wild‐type group. (E) *IMUP* expression was higher in the RAS wild‐type group than in the RAS mutation group. (F) *IMUP* expression was higher in the BRAF‐like group than in the RAS‐like group. Kaplan‐Meier analyses of disease‐free survival based on median (E) or quartile (F) level of *IMUP* expression in the BRAF‐like group from TCGA. Statistical analyses were performed as follows: (A, B): Kruskal‐Wallis test; (C‐F): Mann‐Whitney test. **P < 0.01, ***P < 0.001, ****P < 0.0001

To explore the association between *IMUP* expression and clinicopathological features of PTC patients, we divided PTC tumour samples into the high and low‐expression group according to respective *IMUP* expression median value in the validated and TCGA cohorts. In our validated cohort, *IMUP* expression was correlated with tumour size (*P* = 0.023) and lymph node metastasis (LNM) (*P* = 0.015) (Table 1). In the TCGA cohort, our results showed that histological type (*P* < 0.001), T stage (*P* = 0.004), LNM (*P* < 0.001) and disease stage (*P* = 0.006) have a significant association with *IMUP* expression (Table 2). Then, we evaluated *IMUP* expression in PTC patients with different tumour stages, subtypes, and molecular classification in the TCGA cohort. As illustrated in Figure 2A, most patients with higher disease stages had higher expression of *IMUP* *IMUP* was significantly higher in the classical and columnar variant subtypes than in the follicular subtype (Figure 2B). Moreover, the *IMUP* mRNA levels in patients with LNM were significantly higher than those without LNM (*P* < 0.0001, Figure 2C). According to a previous report, PTC could be mainly classified into BRAF‐like and RAS‐like subtypes based on the driver mutation status and genome profile.^7^ The BRAF‐like PTC driven by BRAFV600E mutation shows the excessive activation of MAPK signalling, while the RAS‐like PTC that driven by RAS and Receptor Tyrosine Kinase exhibits aberrant activation of Phosphoinositide 3‐Kinase signalling.^7^ In our study, we discovered that *IMUP* expression was higher in the BRAF mutation group (Figure 2D, *P* < 0.0001) and RAS wild‐type group (Figure 2E, *P* < 0.0001) than in the respective counterpart group. Based on the classification mentioned above, we found that *IMUP* expression is significantly higher in the BRAF‐like group than the RAS‐like group (*P* < 0.0001, Figure 2F).

**TABLE 1:** The association between *IMUP* expression and clinicopathologic features in the validated cohort

Clinicopathologic features	Cases	High expression (%)	Low expression (%)	χ^2^	*P*
Gender					
Male	15	9 (60.0)	6 (40.0)	1.242	0.216
Female	24	10 (41.7)	14 (58.3)		
Age (y)					
<55	24	13 (54.2)	11 (45.8)	0.742	0.389
≥55	15	6 (40.0)	9 (60.0)		
Neoplasm focus type					
Unifocal	29	15 (51.7)	14 (48.3)	0.409	0.522
Multifocal	10	4 (40.0)	6 (60.0)		
Tumour size (mm)					
≤10	13	3 (23.1)	10 (76.9)	5.132	0.023
>10	26	16 (61.5)	10 (38.5)		
Lymph node metastasis					
Yes	21	14 (66.7)	7 (33.3)	5.867	0.015
No	18	5 (27.8)	13 (72.2)		
Disease stage (AJCC7)					
I + II	24	9 (37.5)	15 (62.5)	3.143	0.076
III + IV	15	10 (66.7)	5 (33.3)		

Abbreviations: AJCC7, American Joint Committee on Cancer Classification, the 7th edition; *IMUP*, immortalization up‐regulated protein.

**TABLE 2:** The association between *IMUP* expression and clinicopathologic features in the TCGA cohort

Clinicopathologic features	Cases	High expression (%)	Low expression (%)	χ^2^	*P*
Gender					
Male	135	70 (51.9)	65 (48.1)	0.253	0.615
Female	367	181 (49.3)	186 (50.7)		
Age (y)					
<55	352	176 (50.0)	176 (50.0)	0.000	1.000
≥55	150	75 (50.0)	75 (50.0)		
Histological type					
Classical	356	202 (56.7)	154 (43.3)	22.253	<0.001
Other types	146	49 (33.6)	97 (66.4)		
Neoplasm focus type					
Unifocal	266	139 (52.3)	127 (47.7)	0.976	0.323
Multifocal	226	108 (47.8)	118 (52.2)		
T stage					
I + II	307	138 (45.0)	169 (55.0)	8.110	0.004
III + IV	193	112 (58.0)	81 (42.0)		
Lymph node metastasis					
Yes	222	140 (63.1)	82 (36.9)	23.641	<0.001
No	229	92 (40.2)	137 (59.8)		
Disease stage (AJCC7)					
I + II	333	152 (45.6)	181 (54.4)	7.561	0.006
III + IV	167	98 (58.7)	69 (41.3)		

Abbreviations: AJCC7, American Joint Committee on Cancer Classification, the 7th edition; IMUP, immortalization up‐regulated protein; TCGA, The Cancer Genome Atlas.

To further explore the prognostic value of *IMUP* in the BRAF‐like group, we performed the survival analyses using Kaplan‐Meier curves from the GEPIA2. Higher *IMUP* expression based on median value was associated with a higher risk of relapse or death in the BRAF‐like subgroup (hazard ratio [HR] = 2.0, *P* = 0.085, Figure 2G). The result was more significant when the classification was based on the quartile value (HR = 3.7, *P* = 0.035, Figure 2H). Thus, *IMUP* overexpression is correlated with more aggressive clinicopathological features and may predict a worse prognosis of PTC patients.

### 3.4 Up‐regulation of *IMUP* increases the risk of LNM in PTC

To examine whether high *IMUP* expression is a major risk factor for LNM, we performed logistic regression analyses. Our results of univariate analysis revealed that the significant variables for LNM consist of higher *IMUP* expression (OR [odds ratio] = 2.542, 95% CI = 1.739‐3.716, *P* < 0.001), classical histological type (OR = 2.370, 95% CI = 1.535‐3.660, *P* < 0.001), female (OR = 0.640, 95% CI = 0.422‐0.972, *P* = 0.036), more advanced disease stage (OR = 3.524, 95% CI = 2.336‐5.316, *P* < 0.001) and T stage (OR = 2.688, 95% CI = 1.820‐3.970, *P* < 0.001). The results of multivariate analysis demonstrated that high expression of *IMUP* (OR = 2.053, 95% CI = 1.365‐3.086, *P = *0.001), classical histological type (OR = 2.657, 95% CI = 1.631‐4.330, *P* < 0.001), more advanced disease stage (OR = 2.792, 95% CI = 1.723‐4.525, *P* < 0.001) and T stage (OR = 1.737, 95% CI = 1.084‐2.784, *P* = 0.022) are factors that have significantly correlation with LNM in PTC (Table 3). These data suggested that high level of *IMUP* expression is an independent predictive factor for LNM.

**TABLE 3:** Univariate and multivariate logistic regression analysis for the risk of lymph node metastasis in the TCGA cohort

Factors	Univariate analysis			Multivariate analysis		
	OR	95% CI	*P*	OR	95% CI	*P*
*IMUP* expression (high vs low)	2.542	1.739‐3.716	<0.001	2.053	1.365‐3.086	0.001
Histological type (classical vs others)	2.370	1.535‐3.660	<0.001	2.657	1.631‐4.330	<0.001
Age (y, ≥55 vs <55)	0.685	0.456‐1.027	0.067		—	
Gender (female vs male)	0.640	0.422‐0.972	0.036	0.769	0.486‐1.219	0.264
Disease stage (AJCC7)	3.524	2.336‐5.316	<0.001	2.792	1.723‐4.525	<0.001
T stage	2.688	1.820‐3.970	<0.001	1.737	1.084‐2.784	0.022
Neoplasm focus type (Mul vs Uni)	1.433	0.985‐2.086	0.060		—	

Abbreviations: AJCC7, American Joint Committee on Cancer Classification, the 7th edition; CI, confidence interval; IMUP, immortalization up‐regulated protein; OR, odds ratio; TCGA, The Cancer Genome Atlas.

### 3.5 Down‐regulation of *IMUP* suppresses proliferation of PTC cell lines

**FIGURE 3:**
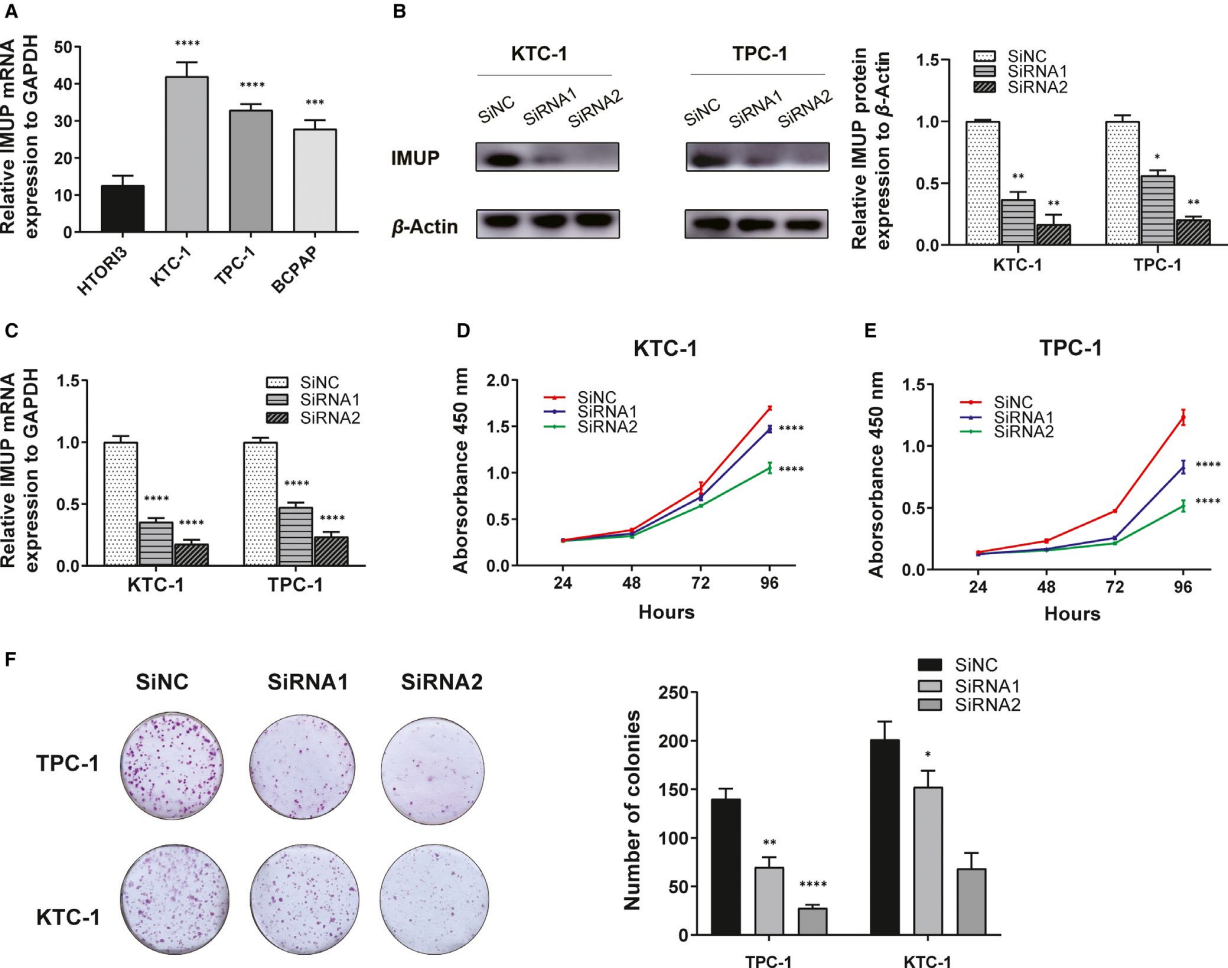
The down‐regulation of *IMUP* suppresses the proliferation and colony formation of PTC cell lines. A, The relative expression of *IMUP* in cell lines (compared with the *GAPDH*) using qRT‐PCR. KTC‐1 and TPC‐1 cells exhibited relatively higher expression. B, The relative protein expression of IMUP (compared with the *β*‐Actin) in KTC‐1 and TPC‐1 cells was significantly lower after being transfected by siRNA1 and siRNA2 that target to *IMUP*. C, The relative mRNA expression of the *IMUP* (compared with the *GAPDH*) in KTC‐1 and TPC‐1 cells was significantly lower after being transfected by siRNA1 and siRNA2 that target to *IMUP*. D and E, CCK‐8 assays of down‐regulated *IMUP* in TPC‐1 and KTC‐1 cell lines. The proliferation of cells that transfected by siRNA1 and siRNA2 was suppressed compared with the corresponding control group (****P < 0.0001 using two‐way ANOVA). F, Colony formation of down‐regulated *IMUP* in TPC‐1 and KTC‐1 cells. The number of colonies was less in transfected cell lines than the control group. *P < 0.05; **P < 0.01; ***P < 0.001; ****P < 0.0001 in comparison with the control group using Student's *t* test

To specify the function of *IMUP*, we started our in vitro study by examining the relative expression level of *IMUP* compared with *GAPDH* in different PTC cell lines. Results showed that KTC‐1, TPC‐1 and BCPAP cell lines express higher *IMUP* than normal thyroid cells (HTORI3) (Figure 3A). We then used two small interfering RNA sequences (SiRNA1 and SiRNA2) to silence the target gene in our selected cell lines with the highest *IMUP* expression (KTC‐1 and TPC‐1); results showed that the relative mRNA and protein expression of *IMUP* was effectively down‐regulated (Figure 3B,C). It was clearly shown in our CCK‐8 assay that the proliferation capacity of *IMUP* down‐regulated cell lines was suppressed (Figure 3D,E), the same as the result of colony formation where the number of IMUP‐knockdown colonies was significantly lesser than the normal ones (Figure 3F). The above results demonstrated that *IMUP* could enhance the proliferation of PTC cells.

### 3.6 Down‐regulation of *IMUP* suppresses migration and invasion of PTC cell lines

**FIGURE 4:**
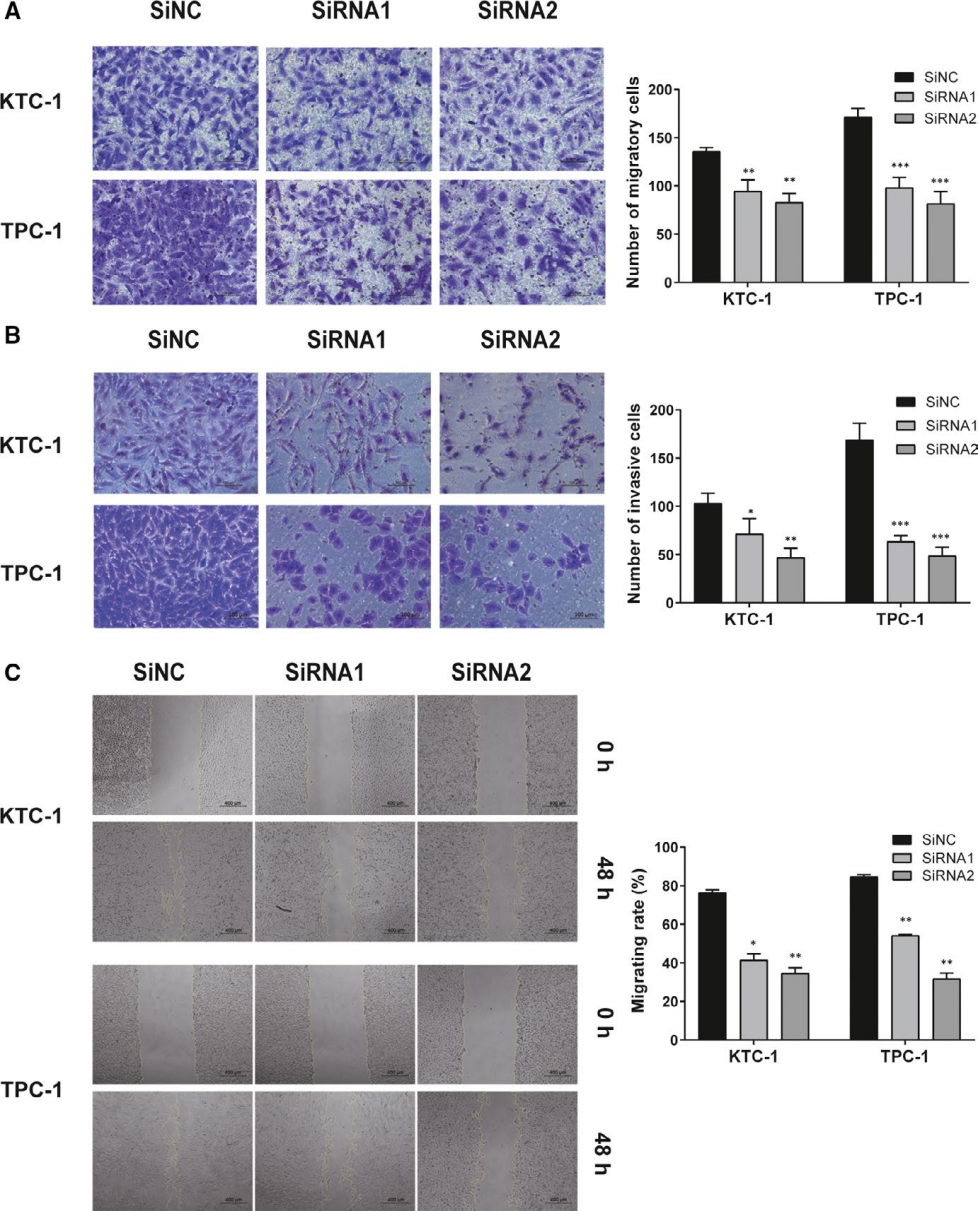
Down‐regulated *IMUP* hinders the migration and invasion of PTC cell lines. A, Transwell migration assay and B, Transwell invasion assay of down‐regulated *IMUP* and corresponding control group. The stained cells were manually counted from five randomly selected fields under the microscope at ×20 magnification. C, Wound healing assay of transfected cells and corresponding control group. The migrating rate (%) = (wound area at 0 h−wound area at 48 h)/wound area at 0 h × 100%. *P < 0.05; **P < 0.01; ***P < 0.001; in comparison with the control group using Student's *t* test

To validate the hypothesis that the expression of *IMUP* would be associated with the metastasis of PTC cell lines, we conducted further experiments. We found that the migratory capacity of down‐regulated *IMUP* PTC cells was inhibited compared with the control groups (Figure 4A); similar results were observed in the invasion experiments (Figure 4B). The scratch tests showed the gaps of IMUP‐knockdown cells were wider after 48 hours compared with the control groups, reconfirming that the ability of migration was inhibited as the migrating rate dropped in the down‐regulated group (Figure 4C). These results indicated that the down‐regulation of *IMUP* suppresses migration and invasion of PTC cell lines.

### 3.7 Down‐regulation of *IMUP* promotes apoptosis and decreases the YAP1/TAZ expression of PTC cell lines

**FIGURE 5:**
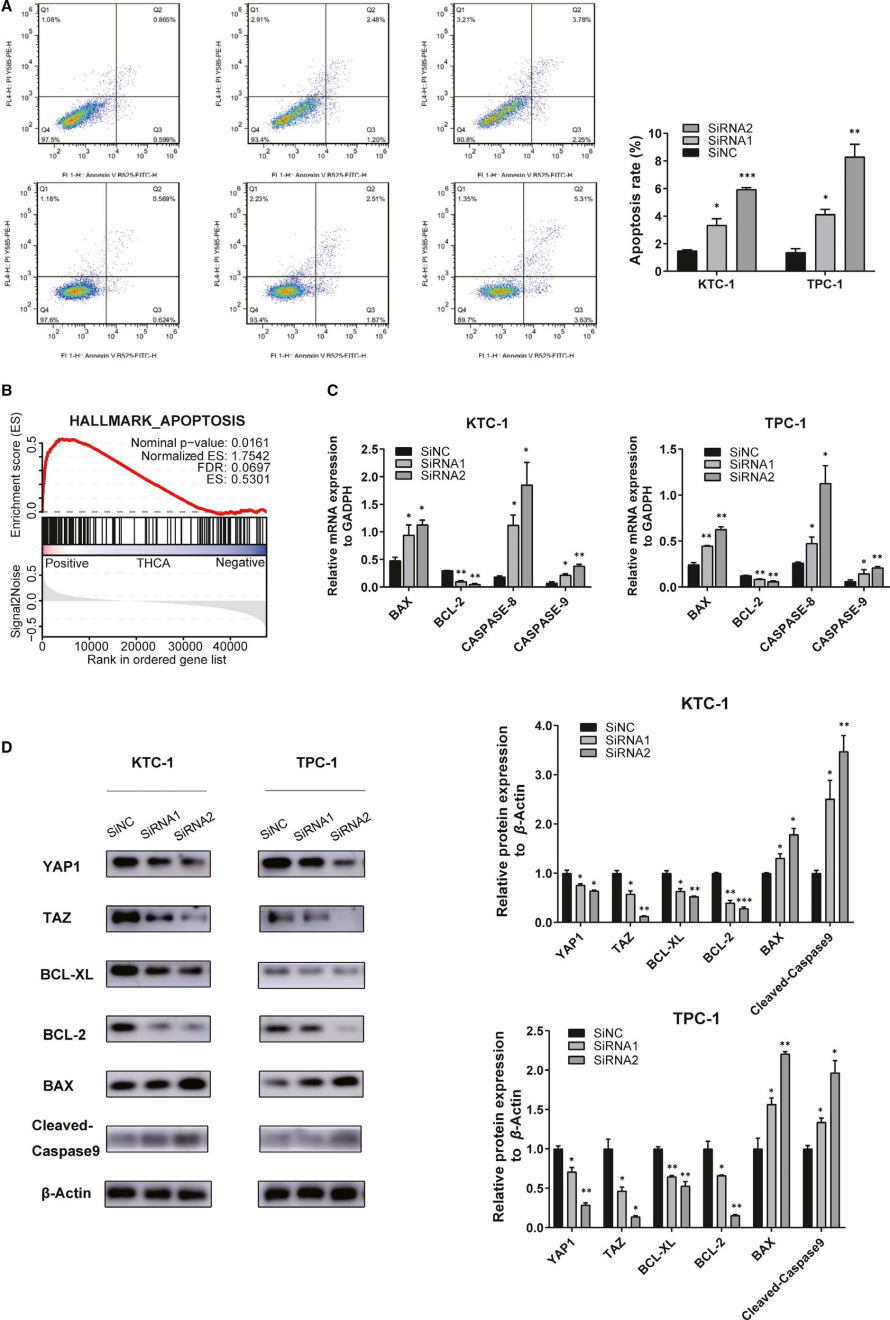
Down‐regulation of *IMUP* induces cell apoptosis and regulates the YAP1/TAZ expression in TPC‐1 and KTC‐1. A, Annexin V/PI assay of *IMUP* down‐regulated PTC cell lines. The proportion of apoptosis is higher in *IMUP* down‐regulated cells than in the control group. Y‐axis: PI staining; X‐axis: Annexin V‐FITC staining. The apoptosis rate was the sum of the statistics of Q2 + Q3. B, GSEA analysis of the correlation between *IMUP* expression and gene signatures of apoptosis. FDR: false discovery rate. C, The qRT‐PCR results showed that silencing *IMUP* decreases the expression of *BCL‐2* and elevates the mRNA expression of *BAX*, *CASPASE‐8* and *CASPASE‐9* in TPC‐1 and KTC‐1. D, The influence of down‐regulated *IMUP* in transfected cells on the levels of YAP1, TAZ, BCL‐XL, BCL‐2, BAX, and Cleaved‐Caspase9 in TPC‐1 and KTC‐1 by Western blotting and corresponding quantitative results. Student's *t* test was used for statistical analyses; *P < 0.05; **P < 0.01; ***P < 0.001

To further explore the influence of IMUP on the tumorigenesis of KTC‐1 and TPC‐1 cell lines, we performed flow cytometry to investigate the apoptosis of transfected cells. We observed increased apoptosis in both KTC and TPC cell lines after silencing *IMUP* (Figure 5A). The apoptosis cells were quantified by the number of early apoptotic cells (Quarter 2) plus the late apoptotic cells (Quarter 3). Then, we analysed expression differences between *IMUP* high‐expression samples and *IMUP* low‐expression samples in the TCGA cohort using GSEA, finding that *IMUP* might be involved in apoptosis‐related pathways (ES = 0.5301, *P* = 0.0161) (Figure 5B). As shown in Figure 5C, in the *IMUP* down‐regulated KTC‐1 and TPC‐1 cells, the relative mRNA expression of *BAX*, *CASPASE‐8* and *CASPASE‐9* was increased while the expression of *BCL‐2* was decreased. Through the literature research, we learned that the Hippo pathway plays a vital role in the regulation of tumour proliferation, migration and apoptosis, therefore, exerting influence on tumour development.^17^, ^18^ YAP1 and TAZ, the major downstream effectors of the Hippo pathway, are negatively regulated by the Hippo kinase cascade.^19^ The results of Western blotting showed that the expression of YAP1 and TAZ was significantly decreased; the protein expression of BAX, Cleaved‐Caspase9 increased while BCL‐XL and BCL‐2 were decreased (Figure 5D). These results suggested that *IMUP* in PTC cells may promote tumour malignant phenotype by affecting the Hippo‐YAP1/TAZ pathway.

### 3.8 The oncogenic role of *IMUP* is partly dependent on YAP1

**FIGURE 6:**
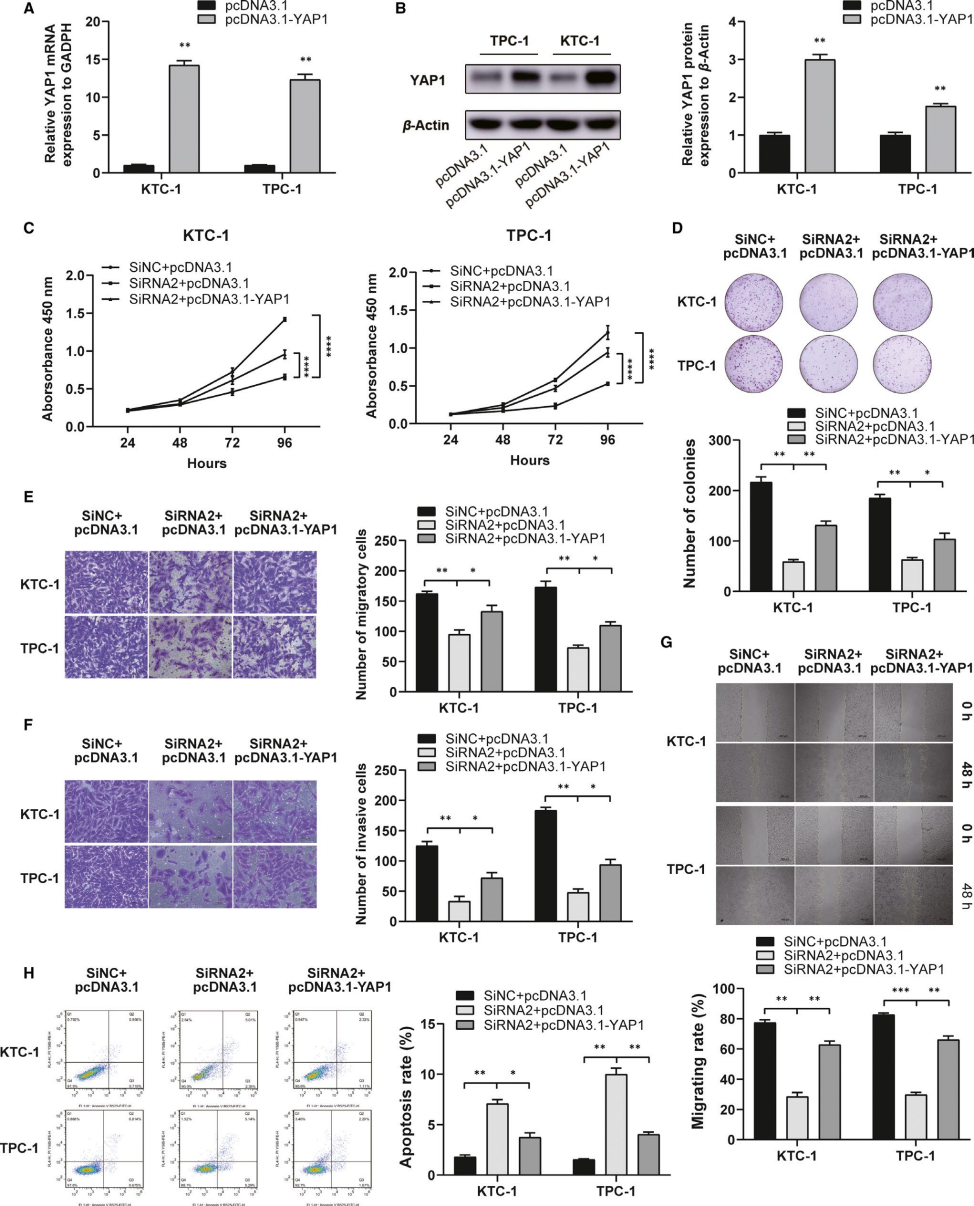
The oncogenic role of *IMUP* is partially dependent on *YAP1* expression. A and B, Compared with the control group, the YAP1 mRNA and protein expression in the pcDNA3.1‐YAP1 group was higher. C and D, CCK‐8 and colony formation assays were conducted to determine proliferative capacities of KTC‐1 and TPC‐1 cells (transfected with SiNC + pcDNA3.1, SiRNA2 + pcDNA3.1, SiRNA2 + pcDNA3.1‐YAP1). E‐G, Transwell and wound healing assays showed that exogenous *YAP1* expression reversed the suppression of migration and invasion caused by *IMUP* silencing. H, Apoptosis assay showed that overexpression of *YAP1* rescues the apoptosis‐promoting effect caused by *IMUP* silencing. Student's *t* test was used for statistical analyses; *P < 0.05, **P < 0.01, ***P < 0.001

According to reports, *YAP1* is a key effector of the Hippo pathway and a cancer‐promoting gene in PTC.^20^, ^21^, ^22^ As the expression of YAP1 was down‐regulated when the *IMUP* was silenced, rescue experiments were employed to explore whether *YAP1* is involved in the effect of *IMUP* on PTC cell progression. The *YAP1* was overexpressed in the TPC‐1 and KTC‐1 using plasmid (pcDNA3.1‐YAP1), and the transfection efficiency was confirmed at RNA and protein levels (Figure 6A,B). The CCK8 assay and colony formation assay showed that the up‐regulation of *YAP1* rescues *IMUP* silencing‐induced inhibition of cell proliferation (Figure 6C,D). The Transwell migration, invasion and wound healing assays indicated that overexpression of *YAP1* partially counteracts the *IMUP* silencing‐mediated effects on migration and invasion in PTC cell lines (Figure 6E‐G). The apoptosis rate of *IMUP*‐knockdown cells was decreased when the expression of *YAP1* was up‐regulated (Figure 6H). These results revealed that *IMUP* promotes PTC cell proliferation, migration and invasion while inhibiting apoptosis via *YAP1*.

## 4 DISCUSSION

In the present study, according to the bioinformatics analysis and literature search, we identified that the gene *IMUP* was one of the most significant DEGs in PTC. We then validated the up‐regulation of *IMUP* in PTC in several datasets and validated cohorts. ROC analysis showed that the expression of *IMUP* could statistically distinguish PTC tumour from normal tissue. Our clinical correlation analyses indicated that high expression of *IMUP* is related to more aggressive clinical manifestations. Moreover, subgroup analysis revealed that the high expression of *IMUP* is associated with worse DFS in the BRAF‐like group. We performed a serial of biological experiments on PTC cell lines; the results demonstrated that down‐regulated *IMUP* suppresses cell proliferation, invasion and migration while increasing the apoptosis of PTC cell lines. Silencing *IMUP* elevated the expression of the pro‐apoptotic proteins (BAX, Cleaved‐Caspase9) but reduced the expression of the anti‐apoptotic proteins (BCL‐2 and BCL‐XL). All results indicated that *IMUP* is an oncogene associated with the occurrence and development of PTC.

In previous reports, the expression of *IMUP* was found to be markedly up‐regulated in many types of cancers, including ovarian epithelial tumours,^23^ endometrial carcinoma,^10^ and lung carcinoma cell lines.^9^ ZY Ryoo et al found that the overexpression of *IMUP* would lead to shortened cell‐cycle in NIH/3T3 mouse fibroblasts, which affects the proliferation rate of cells in vitro.^8^ Jung et al reported in one of their studies that the hypoxia condition could induce the up‐regulation of *IMUP‐2* expression, leading to the apoptosis of HTR‐8/SVneo trophoblast cells, therefore, related to diseases such as pre‐eclampsia.^24^ Their further study proved that the apoptosis in trophoblast cells was mediated by the interaction between hypoxia‐induced down‐regulation of X‐linked inhibitor of apoptosis and up‐regulation of *IMUP‐2*.^25^ However, our study found that the down‐regulation of *IMUP* induces apoptosis in PTC cell lines, which could result from the functional variation of *IMUP* in different pathological processes.

Hippo pathway was first discovered in *Drosophila* and plays a conserved role in the mammal in organ size control, cell proliferation and development, and survival.^26^, ^27^, ^28^ Through a series of kinase cascade via tumour suppressors MST/LATS, the hippo pathway inhibits the bind between YAP/TAZ and TEAD, inhibiting the downstream gene transcriptional activities, eventually hindering tumour proliferation, metastasis and anti‐apoptosis.^29^ The activation of the Hippo pathway could be initiated by cell density sensing, DNA damage along with other signalling molecules.^30^, ^31^ *YAP1* is one of the most important effectors downstream of the Hippo signalling pathway and its aberrant expression that contributes to tumour progression indicates poor outcomes in various cancers.^32^, ^33^, ^34^ It has been reported that the HIPPO pathway is relatively inactive in PTC compared with that in normal thyroid tissues.^35^ Some also reported that *YAP1* is overexpressed and serves as an oncogene that correlates with poor prognosis of PTC patients.^22^, ^36^, ^37^ In the present study, we observed that the down‐regulation of *IMUP* could decrease the protein expression of YAP1 and TAZ. Overexpression of *YAP1* partially rescued the tumour‐suppressing effects caused by *IMUP* silencing, suggesting that *IMUP* might promote tumorigenesis and progression of PTC via the HIPPO‐YAP1 pathway.

However, our work had several shortcomings. First, the number of patients in our validated cohort was relatively limited; an expanded cohort could have offered a more convincing result. Secondly, the specific way of IMUP‐HIPPO‐YAP1 interaction and the relationship between *IMUP* and other components of the HIPPO pathway remain to be elucidated. Finally, we still need animal experiments to validate the function of *IMUP* in PTC further.

In summary, we found that the expression of *IMUP* is up‐regulated in PTC and positively associated with LNM and worse clinical outcomes. Silenced *IMUP* could suppress the proliferation, migration, and invasion, and promote apoptosis of PTC cells. *IMUP* may promote progression of PTC tumour cells via the Hippo‐YAP1 pathway. These findings could provide a new marker and target gene in the treating regimen of TC.

## CONFLICT OF INTEREST

The authors declare that they have no conflict of interest.

## AUTHOR CONTRIBUTION

**Lizhi Lin:** Investigation (equal); supervision (equal); writing – original draft (lead); writing – review and editing (lead). **Jialiang Wen:** Data curation (lead); methodology (equal); validation (equal); writing – original draft (equal); writing – review and editing (equal). **Bangyi Lin:** Data curation (equal); investigation (equal); validation (equal). **Adheesh Bhandari:** Data curation (supporting); software (supporting); validation (supporting); writing – review and editing (equal). **Danni Zheng:** Data curation (supporting); investigation (supporting); software (supporting); validation (equal); writing – review and editing (supporting). **Linguo Kong:** Formal analysis (equal); resources (supporting). **Yinghao Wang:** Formal analysis (equal); funding acquisition (supporting); supervision (supporting). **Ouchen Wang:** Conceptualization (equal); funding acquisition (equal); supervision (supporting). **Yizuo Chen:** Conceptualization (equal); funding acquisition (lead); project administration (equal); supervision (equal).

## Supporting information

Fig S1

Table S1

## Data Availability

The sources of public datasets supporting the conclusions of this study are shown in this article. Other raw data are available on the main electronic data storage system of the First Affiliated Hospital of Wenzhou Medical University.
