# Supplementary material for: Immortalization up‐regulated protein promotes tumorigenesis and inhibits apoptosis of papillary thyroid cancer
Source: J Cell Mol Med. 2020 Oct 23;24(23):14059–72. doi: 10.1111/jcmm.16018 (PMC7754061; doi:10.1111/jcmm.16018)
Supplement: Supplementary file 1 — Fig S1 [file JCMM-24-14059-s001.docx]

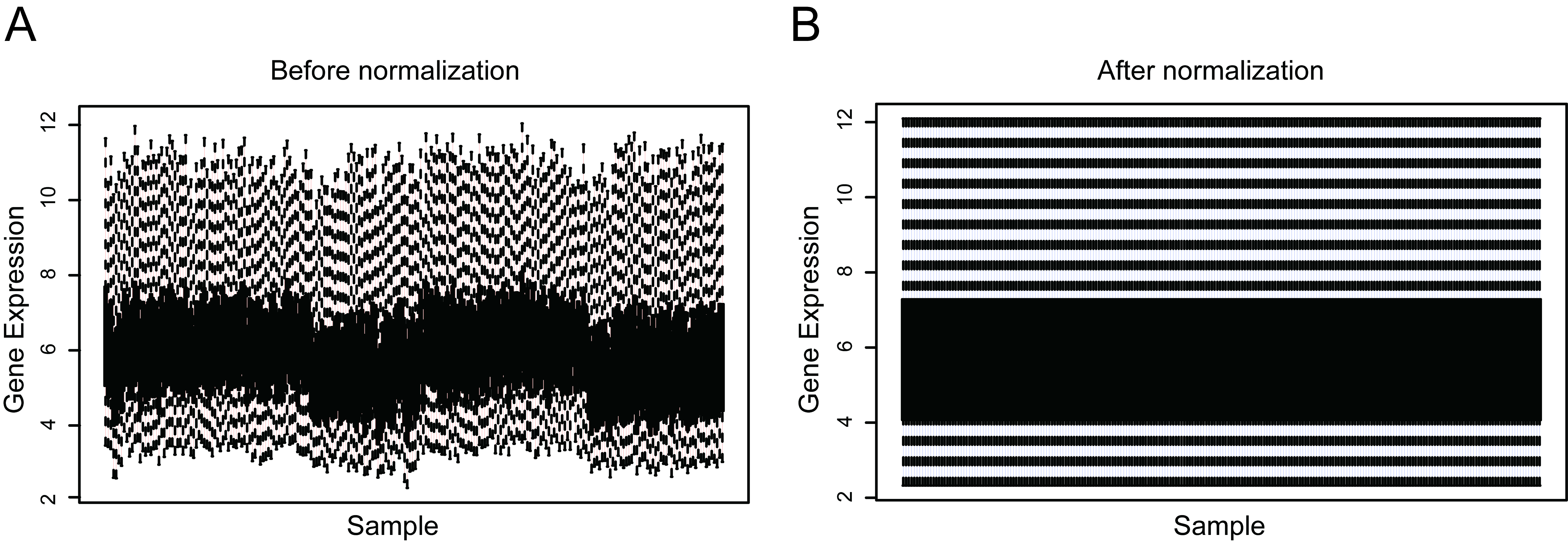


**Figure S1: Integrated data of gene expression in GSE33630, GSE60542, GSE35570 datasets** The data before normalization (A) and after normalization (B)
