# Supplementary material for: Immortalization up‐regulated protein promotes tumorigenesis and inhibits apoptosis of papillary thyroid cancer
Source: J Cell Mol Med. 2020 Oct 23;24(23):14059–72. doi: 10.1111/jcmm.16018 (PMC7754061; doi:10.1111/jcmm.16018)
Supplement: Supplementary file 2 — Table S1 [file JCMM-24-14059-s002.docx]

| **Table S1.** Information of GEO series mined in this analysis | | | |  |
| --- | --- | --- | --- | --- |
| GEO series | Contributor(s) | PTC Tumor sample | Non-tumor control | Platform |
| GSE33630 | Tomas G, Vincent D | 49 | 45 | GPL570, Affymetrix Human Genome U133 Plus 2.0 Array |
| GSE60542 | Tarabichi M, Saiselet M,*et al.* | 28 | 27 | GPL570, Affymetrix Human Genome U133 Plus 2.0 Array |
| GSE35570 | Jarzab B | 32 | 51 | GPL570, Affymetrix Human Genome U133 Plus 2.0 Array |
| GSE50901 | Barros Filho MC, Marchi FA | 61 | 4 | GPL13607, Agilent-028004 SurePrint G3 Human GE 8x60K Microarray |
| Abbreviations: GEO, Gene Expression Omnibus; PTC, papillary thyroid cancer | | | | |
|  |  |  |  |  |
